# Editorial: Medical Image Perception: How Much Do We Understand It?

**DOI:** 10.3389/fpsyg.2017.02072

**Published:** 2017-11-28

**Authors:** Tim Donovan, Damien Litchfield, Trevor J. Crawford

**Affiliations:** ^1^Medical and Sport Sciences, University of Cumbria, Lancaster, United Kingdom; ^2^Department of Psychology, Lancaster University, Lancaster, United Kingdom; ^3^Department of Psychology, Edge Hill University, Ormskirk, United Kingdom

**Keywords:** medical image perception, holistic processing, radiology, expert performance, stack viewing

Although there have been great advances in understanding radiological expertise, in particular the type of errors made, there is still a limited understanding of the underlying processes that are involved in accurate medical image perception and to date the medical image perception and vision science literatures have largely developed separately. In this Research Topic, therefore, we set out six clear aims, and despite only having four accepted papers, because they have taken four distinct approaches to the Topic we have met most of the aims. This is an important area of research as demonstrated by the concurrent special issues relating to medical image perception such as this Topic (*Frontline Learning Research and Cognitive Research: Principles and Implications*). All calls make the point that this is an opportunity to bring together previously separate lines of research to gain greater understanding about a subject which has real world implications for medical health care.

Our first two aims were to review the current state of play in our understanding of medical image perception, and determine the intersection between vision science and the radiological task. In addressing this Sheridan and Reingold have written a very clear and succinct review which looks at the empirical evidence relating to holistic processing and how the medical image is perceived. The advantage of a review such as this is that one can step back and gain an overall perspective on what empirical support currently exists for the global-focal search model (Nodine and Kundel, 1987) and the two-stage detection model (Swensson, 1980), as well as related theoretical perspectives developed in psychology (e.g., Torralba et al., 2006; Wolfe et al., 2011). An experimental psychology approach is vital to test assumptions, such as the importance of holistic processing in expert performance, and in this respect Sheridan and Reingold highlight the work of Litchfield and Donovan (2016) using a gaze contingent paradigm following a brief (250 ms) preview of a medical image or mask. They did not find a benefit of preview for experts, and Sheridan and Reingold indicate that this finding can be reconciled with models which suggest that global processing extends beyond the initial glimpse.

The third aim was to map out the factors contributing to radiological errors. The contributions by Crowe et al. and Nakashima et al. provide some interesting insights and both have compared novices to experts. Most early medical image perception research just tested radiologists as novice-expert comparisons were simply not perceived as being relevant as novices would not be used to read imaging studies. It is, however, fundamental not just to show experts are better, but to gain insight into experts underlying representations and how they develop. Both contributions have also used datasets from cross-sectional imaging studies where observers can scroll through image stacks. This is important because the radiological task is very domain specific and the majority of previous research has used test banks consisting of 2D images such as chest radiographs, whereas a considerable proportion of current examinations now involve looking at large datasets of stack images from CT and MRI. Nakashima et al's intriguing finding that the temporal location of lesions, i.e., whether they occur early in the image sequence, has a significant effect on the performance of novices, but not of radiologists. The authors suggest that the transient signal of the lesion appearance is masked by a global apparent motion onset. Although, as previously stated, novices would not be used to interpret medical images it does suggest that radiologists are better because they have a good target template and can extract global information. The study by Crowe et al. investigating tumor delineation is a very relevant issue as it is important to know if tumors are growing or shrinking in response to treatment. There has always been an awareness of intra and inter observer variability in these type of radiological tasks and indeed low inter-observer agreement was replicated in this study. The lack of an expertise effect is possibly due to the heterogeneity of the expertise group, but the finding that experts are more consistent at peripheral slices as the tumor boundary is clinically very important.

Our fourth aim was to review evidence-based studies on the guidance for enhancing training and practice. As medical image educators, we do not know which is the best way to develop accurate anatomic-pathologic schemas which lead to the perceptual differentiation of abnormalities. Kok et al. in their review discuss diagnostic reasoning, cognitive schemas, and study strategies. It is striking how much of a gap there is in the literature of good theory-driven studies of specific interventions, and particularly how non-analytical reasoning develops or should be trained. Nevertheless, Kok et al. make some useful recommendations as to how medical image interpretation should be taught and the importance of developing appropriate cognitive schemas.

Unfortunately, there were no submissions relating to the 5th aim which is the relationship between computer aided detection/diagnosis (CAD/CADx) and human expertise. This is a pity as there are currently rapid developments in radiology with the introduction of artificial intelligence (AI) and big data analytics; indeed, the introduction of a new term “radiomics” relating to the computer analysis of medical images has become prevalent (Gillies et al., 2016). It is unknown how this will impact on the human observer and the radiological task. Similarly, there were no submissions to address our 6th aim of evaluating recent research on imitation and observational learning for performance on perceptual detection tasks. Given the challenges facing radiology, it seems that this is not currently a high priority area for research.

This Research Topic has demonstrated the importance of taking a multi-disciplinary approach to understanding expert performance and the under-lying processes in real-world tasks such as medical image interpretation; however, in such a rapid technologically developing domain such as radiology it is likely research will always be trying to catch up in order to inform the efficacy of new developments in the radiological task.

## Author contributions

TD drafted the article, all authors revised it critically for important intellectual content, and approved it for publication.

### Conflict of interest statement

The authors declare that the research was conducted in the absence of any commercial or financial relationships that could be construed as a potential conflict of interest.
